# Transcription factor signal transducer and activator of transcription 6 (STAT6) is an inhibitory factor for adult myogenesis

**DOI:** 10.1186/s13395-021-00271-8

**Published:** 2021-05-29

**Authors:** Mitsutoshi Kurosaka, Yuji Ogura, Shuichi Sato, Kazuhisa Kohda, Toshiya Funabashi

**Affiliations:** 1 Department of Physiology, St. Marianna University School of Medicine, Kawasaki, Kanagawa 216-8511 Japan; 2 School of Kinesiology, The University of Louisiana at Lafayette, Lafayette, LA USA; 3 New Iberia Research Center, The University of Louisiana at Lafayette, New Iberia, LA USA

**Keywords:** Myotube, Myoblast fusion, Differentiation, Primary myoblast, Interleukin-4

## Abstract

**Background:**

The signal transducer and activator of transcription 6 (STAT6) transcription factor plays a vitally important role in immune cells, where it is activated mainly by interleukin-4 (IL-4). Because IL-4 is an essential cytokine for myotube formation, STAT6 might also be involved in myogenesis as part of IL-4 signaling. This study was conducted to elucidate the role of STAT6 in adult myogenesis in vitro and in vivo.

**Methods:**

Myoblasts were isolated from male mice and were differentiated on a culture dish to evaluate the change in STAT6 during myotube formation. Then, the effects of STAT6 overexpression and inhibition on proliferation, differentiation, and fusion in those cells were studied. Additionally, to elucidate the myogenic role of STAT6 in vivo, muscle regeneration after injury was evaluated in STAT6 knockout mice.

**Results:**

IL-4 can increase STAT6 phosphorylation, but STAT6 phosphorylation decreased during myotube formation in culture. STAT6 overexpression decreased, but STAT6 knockdown increased the differentiation index and the fusion index. Results indicate that STAT6 inhibited myogenin protein expression. Results of in vivo experiments show that STAT6 knockout mice exhibited better regeneration than wild-type mice 5 days after cardiotoxin-induced injury. It is particularly interesting that results obtained using cells from STAT6 knockout mice suggest that this STAT6 inhibitory action for myogenesis was not mediated by IL-4 but might instead be associated with p38 mitogen-activated protein kinase phosphorylation. However, STAT6 was not involved in the proliferation of myogenic cells in vitro and in vivo.

**Conclusion:**

Results suggest that STAT6 functions as an inhibitor of adult myogenesis. Moreover, results suggest that the IL-4-STAT6 signaling axis is unlikely to be responsible for myotube formation.

**Supplementary Information:**

The online version contains supplementary material available at 10.1186/s13395-021-00271-8.

## Background

The skeletal muscle, which constitutes 40% of human body mass, is indispensable for locomotion, respiration, and metabolism [1, 2]. The skeletal muscle comprises numerous myofibers, each of which contains multiple postmitotic myonuclei. During the formation of multinucleated myofibers in adults, resident myogenic stem cells undergo a unique process called myogenesis [3–5]. Upon request, myogenic stem cells are activated and committed to differentiation. The activated myogenic stem cells (i.e., myoblasts) subsequently fuse together or with (nascent) myotubes to form mature myofibers [3, 6–9]. Obstruction of myogenesis inhibits proper muscle regeneration after injury, leading to decline of skeletal muscle function [3–5]. Many molecules have been presently identified as triggering and coordinating myogenic differentiation, fusion, and maturation of myofibers [8, 10, 11]. Nevertheless, the mechanisms have not been fully elucidated.

The signal transducer and activator of transcription 6 (STAT6) plays a fundamentally important role in immune cells’ cellular function [12, 13]. For instance, STAT6 is involved in T cell proliferation [14]. Also, its activation prevents apoptotic cell death in B cells [15]. It is also involved in the fusion of macrophages to generate multinucleated giant cells in response to inflammation [16]. Recent studies have also demonstrated the involvement of STAT6 in microglial activation in the brain tissue [17]. Nevertheless, the role of STAT6 in peripheral tissues remains unclear.

Earlier studies have revealed STAT6 as an important target of interleukin (IL)-4 in nonmuscle cells [12, 13, 17]. After IL-4 stimulation, STAT6 gets activated by phosphorylation and functions as a transcription factor to promote context-dependent gene expression [12, 13, 17]. In this regard, IL-4 is an essential molecule for myogenesis. Studies have demonstrated that the nuclear factor of activated T cells 2 (NFATc2), a calcium-sensitive transcription factor, specifically localizes nascent myotubes and stimulates IL-4 secretion during myoblast differentiation [18–21]. The secreted IL-4 binds to IL-4 receptor alpha (IL-4Rα) on the surrounding myoblasts to promote the fusion of those myoblasts with nascent myotubes [21]. These results suggest that STAT6 is also involved in myotube formation under the control of IL-4. Nevertheless, the function of STAT6 at any stage of myogenesis remains unknown. Therefore, this study was designed to elucidate whether STAT6 can be implicated in adult myogenesis in vitro and in vivo.

## Methods

### Animals

Wild-type (WT) male C57BL/6J mice were purchased from SLC Inc. (Hamamatsu, Shizuoka, Japan). R26-CAG-LoxP-monomeric teal fluorescent protein 1 (mTFP1) mice (B6;129S6-Gt (ROSA)26Sor^tm1.1(CAG-mTFP1)Imayo^) were obtained from RIKEN (ID: RBRC05147; Tsukuba, Ibaraki, Japan) [22]. Pax7-CreER^T2^ (B6.Cg-Pax7^tm1(cre/ERT2)Gaka^/J) mice were purchased from The Jackson Laboratory (ID: 017763; ME, USA) [23]. Tamoxifen-inducible muscle stem cell-specific mTFP1-expressing mice were generated by crossing R26-CAG-LoxP-mTFP1 and Pax7-CreER^T2^ mice. STAT6-knockout (KO) mice (B6;129P2-Stat6<tm1Aki>/AkiRbrc) were obtained from RIKEN (ID: RBRC00958) [13]. Animals were maintained in an animal facility (25°C, 55% relative humidity, lights on 0600–1800 h). All animals had a BL/6 genetic background and were used at 7–8 weeks of age.

### Cell culture

Primary myoblasts were isolated from the hind limb muscles of WT, STAT6-KO, and muscle satellite cell-specific mTFP1-expressing mice as described in earlier reports of the literature [24, 25]. The expression of mTFP1 was induced by tamoxifen injection (2 mg/animal, four consecutive days) at 1 week before isolation. After purification by preplating, myoblasts were maintained in growth medium (GM, 20% fetal bovine serum, 1% penicillin/streptomycin, and Ham’s F-10; Life Technologies Inc., CA, USA). The medium was supplemented with 5 ng/mL basic fibroblast growth factor (Peprotech Inc., NJ, USA). When cells attained approximately 90% cellular confluency, fusion of the myoblasts was induced by switching from GM to differentiation medium (DM, 2% house serum, 1% penicillin/streptomycin, and Dulbecco’s modified Eagle’s medium; Life Technologies Inc.).

### Adenovirus construction and infection

Adenoviruses carrying mouse STAT6 were generated (AdEasy Adenoviral Vector System; Agilent Technologies Inc., CA, USA) as described in the literature [25]. Mouse STAT6 was amplified from mouse cDNA and ligated into the vector (RedTrack-CMV; Addgene, MA, USA) using the *KpnI* and *XbaI* sites. The resulting AdTrack-CMV-STAT6 plasmid was linearized with PmeI; then, it was cotransformed into *Escherichia coli* BJ5183 cells with the pAdEasy-1 plasmid. Clones undergoing AdTrack–Adeasy recombination were selected with kanamycin and were confirmed by enzyme digestion. The recombinant plasmid was linearized with PacI and was transfected into the Adeno-X cell line (Clontech, Manassas, VA, USA) using Lipofectamine 2000 Transfection Reagent (Life Technologies Inc.) packaging into active virus particles. The produced viruses (adenoviral-RFP-STAT6: Ad-STAT6 or adenoviral-RFP-empty: Ad-Ctrl) were amplified further by serial passage to concentrate. At 70% confluence, Adeno-X cells were infected with the virus and were maintained for 72–96 h. The viral titer was found using an RFP-positive cell number per field. The number of infectious units per milliliter for each well were calculated as (infected cells/field) × (fields/well)/virus volume (mL) × dilution factor. For adenovirus infection, myoblasts (1 × 10^6^) were plated and then infected with either a STAT6 vector (Ad-STAT6) or an empty vector (Ad-Ctrl) using the same concentration of infectious units for 6 h. After the infection period, the infected myoblasts were washed carefully and were then maintained in GM for 48 h. Our adenoviral infection had infection efficiency of nearly 100%.

### Short hairpin RNA (shRNA)

Procedures using shRNA were conducted as described in an earlier report [25]. The pLKO.1-mCherry-puro plasmid was provided by Dr. Renzhi Han (The Ohio State University Wexner Medical Center, OH, USA). Target siRNA sequences for mouse STAT6 (GGTTCAGATGCTTTCTGTTAC) were designed using BLOCK-iT RNAi Designer (Life Technologies Inc.). After the synthesized siRNA oligonucleotides (Integrated DNA Technologies, Inc., Coralville, IA, USA) were annealed, they were inserted into the plasmid using AgeI and EcoRI sites. The appropriate plasmid was amplified using a standard bacterial culture. Then, the siRNA sequence was validated for the knockdown of STAT6 mRNA in preliminary experiments. Control cells were transfected with the backbone plasmid harboring the scramble sequence (CCTAAGGTTAAGTCGCCCTCG).

### Plasmid delivery to myoblasts

Plasmid DNA was transfected by electroporation (1500 V, 10 ms, three pulses) using a transfection system (Neon; Life Technologies Inc.) as described for an earlier study [26]. Our electroporation procedure routinely achieved 70–80% transfection efficiency at 24 h post-transfection [26].

### IL-4 treatment

Recombinant IL-4 (R&D Systems, Minneapolis, MN, USA) was prepared and used similarly to our earlier study [24]. After IL-4 (10 ng/mL) was added to the culture medium in WT and STAT6-KO cells, it was incubated for 48 h. Saline was used as the vehicle.

### Evaluation of fusion of myoblasts to nascent myotubes in a cell mixing experiment

For the cell mixing experiment, we used adenoviral infection to achieve the highest degree of efficiency. The myoblasts isolated from muscle satellite cell-specific mTFP1^+^ and WT mice were grown in GM. Based on procedures used for an earlier study [21], mTFP1^+^ myoblasts were seeded in 24-well plates (0.25 × 10^5^ cells per well) in DM for 48 h to induce myotube formation and to allow estimation of myotube–myoblast fusion. The original protocol used 24 h for pre-DM incubation [21], but 48 h was found to be necessary to form visible myotubes under our experimental conditions. Simultaneously, WT myoblasts were infected with either Ad-STAT6 or Ad-Ctrl in GM for 6 h. After the cells were washed, the well was replenished with fresh GM. The cells were then maintained until the following day. After forming mTFP1^+^ myotubes, Ad-STAT6 or Ad-Ctrl RFP^+^ myoblasts were transferred into the plate to fuse the infected myoblast to mTFP1^+^ myotubes (0.5 × 10^5^ cells per well). They were maintained for a further 48 h. Then, the fusion index and the number of unfused cells were studied by immunocytochemistry.

### Immunocytochemistry

Cells were prepared for immunocytochemistry as described earlier [24]. Primary antibodies specific to the myosin heavy chain (MyHC, MF-20, 1:50, DSHB) and secondary antibodies conjugated with Alexa Fluor 488 (Life Technologies Inc.) were used for staining. Myonuclei were stained with DAPI. Images of stained cells were captured using a microscope (BZ-9000; Keyence Co.) and were analyzed using Fiji software [27]. The fusion index was defined as the ratio of the number of nuclei in myotubes to the number of nuclei in each image. Myonucleus numbers of the myosin^+^ cells were also found. For each experiment, three randomly captured images were analyzed per sample.

### Muscle injury

Into the tibialis anterior (TA) muscle of the left leg of each mouse, 100 μl of 10 μM cardiotoxin (Latoxan, Valence, France) dissolved in saline was injected [24]. Saline was injected into the TA muscle of the right leg as a vehicle. At 5 days post-injection, both TA muscles were excised for analyses.

### Morphological analysis

Frozen TA muscles were kept below −20°C and were cut using a cryostat (Leica Microsystems GmbH, Wetzler, Germany). Sections were stained with hematoxylin and eosin. Images were captured using a microscope (BZ-9000). The cross-sectional area (CSA) was analyzed using Fiji software. The size distribution was evaluated. The average number of central nuclei in regenerated myofibers of the TA muscle was also evaluated for fusion efficiency during regeneration.

### Immunohistochemistry

Cryosections (9-μm thickness) were made from injured and intact TA muscle. They were fixed with 4% paraformaldehyde for 15 min at 25°C. After washing with phosphate-buffered saline (PBS), the sections were blocked with a blocking buffer containing 3% BSA, 5% goat serum, and 0.5% Triton-X for 30 min. After several washes with PBS, the sections were incubated with M.O.M. blocking reagent for 45 min at 25°C (Vector Laboratories Inc., Burlingame, CA, USA). After washing with PBS, they were incubated with primary antibody against Pax7 (1:10, clone Pax7; DSHB, University of Iowa, Iowa City, IA), myogenin (1:10, BD Pharmingen, San Jose, CA, USA), and polyclonal anti-laminin (1:200; Sigma Chemical Co., St. Louis, MO, USA) in staining solution (Can get signal hist A; Toyobo Co. Ltd., Osaka, Osaka, Japan) for 90 min at 25°C. After washing with PBS, the slides were incubated with Alexa Fluor 488 (antirabbit)-conjugated and 568 (antimouse)-conjugated secondary antibody (1:1,000, Thermo Fisher Scientific K.K.) for 30 min at 25°C followed by incubation with DAPI solution (0.01 mg/mL in PBS) for 1 min. After washing, the slides were mounted using a fluorescence medium (Aqua-Poly/Mount; Polysciences Inc., Warrington, PA, USA) and were visualized using a digital microscope (BZ-X9000) and were analyzed using Fiji software.

### Western blotting

Samples were homogenized in an ice-cold buffer (50 mM Tris-Cl, 200 mM NaCl, 50 mM NaF, 0.3% NP-40, pH 8.0) with protease and phosphatase inhibitors (Nacalai Tesque Inc., Chukyo-ku, Kyoto, Japan). The protein concentration was measured using the bicinchoninic acid (BCA) method (BCA assay kit; Thermo Fisher Scientific K.K.). An equal amount of protein was separated using standard SDS-PAGE and was transferred to a PVDF membrane. The membrane was blocked with a blocking reagent (Blocking One; Nacalai Tesque Inc.) and was incubated with primary antibodies. The primary antibodies used were phosphorylated Tyr^641^-STAT6 (1:1000, #9362; Cell Signaling Technology Inc., MA, USA), STAT6 (1:2000, #6778; Cell Signaling Technology Inc.), phosphorylated Thr^180^/Tyr182-p38 Mitogen-activated protein kinase (MAPK; 1:1000, #4511; Cell Signaling Technology Inc.), p38 MAPK (1:1000, #9212; Cell Signaling Technology Inc.), and glyceraldehyde-3-phosphate dehydrogenase (GAPDH; 1:2000, #2118; Cell Signaling Technology Inc.). Luminescence signals by ECL reagent (Bio-rad Laboratories Inc., Hercules, CA, USA) were captured using an imaging system (LAS-4000; Fujifilm Corp., Minato-ku, Tokyo, Japan). Densitometry analysis was conducted using Fiji software.

### Quantitative reverse transcription—polymerase chain reaction (QRT-PCR)

Details of the protocol have been described in an earlier report [24]. Total RNA from the cells was isolated using RNA extraction reagent (Sepasol-RNA I Super G; Nacalai Tesque Inc.) and RNA mini-columns (FATRK 001; Favorgen Biotech Corp., Ping-Tung, Taiwan) according to the manufacturers’ protocols. The first-strand cDNA for PCR was generated using a commercially available kit (FSQ-301; Toyobo Co. Ltd.). Quantification of mRNA expression was performed using a real-time PCR system (Step One Plus; Life Technologies Japan Ltd., Minato-ku, Tokyo) with Syber green master mix reagent (QPS-101; Toyobo Co. Ltd.). For delta–delta Ct analysis, β-Actin or GAPDH mRNA was used as an internal reference. The primer sequences used for this study are presented in the Supplementary Table.

### Indirect cell number measurement

Adenovirus-infected cells were grown for 24 and 48 h in GM. Cells at each time point were counted (cell counting kit-8 #CK04; Dojindo Laboratories, Kamimashiki-gun, Kumamoto, Japan) according to the manufacturer’s instructions. The indirect cell number was expressed with absolute absorbance at 450 nm (Multiskan MS; Life Technologies Inc.). The proliferation rate was calculated as the cell number at 48 h divided by the cell number at 24 h [26].

### Statistical analyses

Data are presented as mean ± standard deviation (SD). One-way analysis of variance (ANOVA) followed by the Tukey–Kramer multiple comparisons test was used to assess multiple group data. Unpaired *t* tests were used for comparisons between the two groups. All analyses were performed using software (Prism v.8.0; GraphPad Software Inc., CA, USA). Significance was inferred for *p* < .05.

## Results

### STAT6 is deactivated during myotube formation, but IL-4 stimulates STAT6 activity

**Fig. 1:**
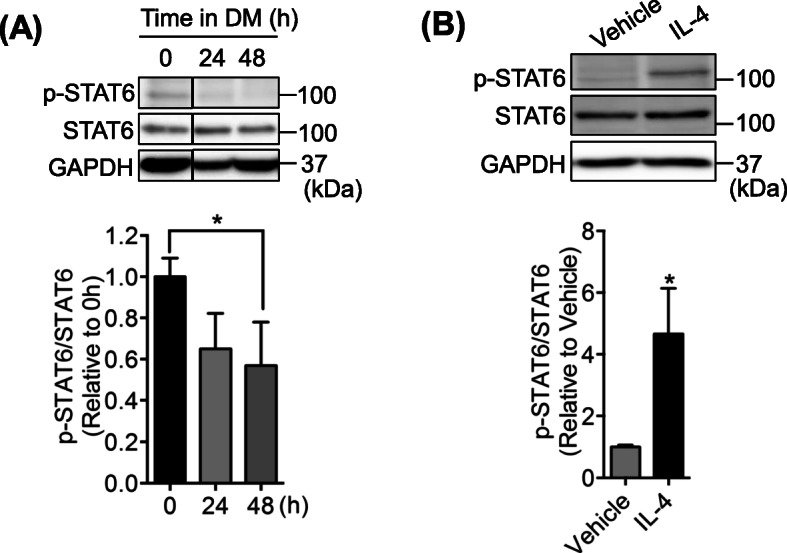
STAT6 was deactivated during myogenesis and IL-4 increased STAT6 phosphorylation. **a** pSTAT6 expression during differentiation. Representative images show Western blot bands for phosphorylated STAT6 (p-STAT6), STAT6, and glyceraldehyde-3-phosphate dehydrogenase (GAPDH) *n* = 3. *p < .05 vs. 0 h. **b** Phosphorylation of STAT6 after IL-4 treatment in cultured myotubes. Representative images of Western blot bands for p-STAT6, STAT6, and GAPDH. *n* = 3. *p < .05 vs. 0 h. Data are presented as mean ± standard deviation (SD)

We first isolated myoblasts from WT mice and incubated the cells in DM for 24 and 48 h myotube formation to investigate phosphorylated-STAT6 (p-STAT6) (Fig. 1a). Results showed that p-STAT6 expression decreased at 24 h after adding DM, which was significant at 48 h. We next examined the effects of IL-4 on STAT6 activation. After the cells were treated with IL-4 during DM incubation for 48 h, we assessed the p-STAT6 expression in those cells. Results showed that p-STAT6 had been increased significantly by IL-4 treatment, indicating that STAT6 is a downstream target for IL-4 during myotube formation (Fig. 1b). These results indicate that STAT6 activity is decreased during normal myogenesis, where endogenous IL-4 is expected to stimulate STAT6 activation [21, 24].

### STAT6 overexpression impairs myoblast fusion

**Fig. 2:**
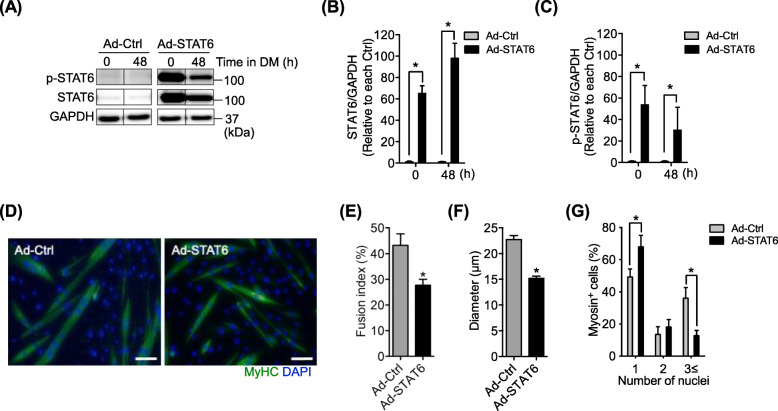
STAT6 overexpression impaired myoblast fusion. **a** Representative images of Western blot bands for p-STAT6 and GAPDH. Myoblasts were infected with Ad-Ctrl or Ad-STAT6 adenovirus vector in growth medium (GM). After 24 h, the medium was replaced with a differentiation medium and maintained for 48 h. The cells were then used for Western blot and immunocytochemical analysis. **b** Relative expression of total STAT6. *n* = 6. *p < .05 vs. Ad-Ctrl. **c** Relative expression of phosphorylated STAT6. *n* = 6. *p < .05 vs. Ad-Ctrl. **d** Representative immunostained myotubes positive for MyHC (green). Nuclei were stained with DAPI (blue). Scale bar = 50 μm. **e** Calculation of the fusion index in the Ad-Ctrl and Ad-STAT6 treatments. **f** Diameters of myotubes in the Ad-Ctrl and Ad-STAT6 treatments. **g** Percentages of myosin-positive cells with one, two, and three or more nuclei. *n* = 6 in each group. *p < .05 vs. Ad-Ctrl. Data are presented as mean ± SD

After finding that STAT6 can be activated by IL-4 during myotube formation, we aimed to clarify whether STAT6 can be involved in myogenesis. Because IL-4 is known to contribute to myoblast fusion, we first specifically examined the link between STAT6 and myoblast fusion. STAT6 was overexpressed in myoblasts by the adenoviral vector. At 24 h after infection, the myoblasts were induced to differentiate by the DM for 48 h. Western blot results confirmed that STAT6 protein expression increased significantly (Fig. 2a and b), indicating that STAT6 was induced successfully. Phosphorylated-STAT6 levels also exhibited the same trend as those of total STAT6 protein (Fig. 2c). We performed immunohistochemical analysis to examine myotube formation (Fig. 2d). The fusion index (Fig. 2e) and diameter (Fig. 2f) of cells overexpressing STAT6 were significantly lower than those of control cells. STAT6 overexpression increased the percentage of myosin-positive cells possessing a single nucleus significantly but decreased the percentage of cells possessing three or more nuclei (Fig. 2g). These results indicate that overexpression of STAT6 impairs myoblast fusion.

### STAT6 knockdown improves myoblast fusion

**Fig. 3.**
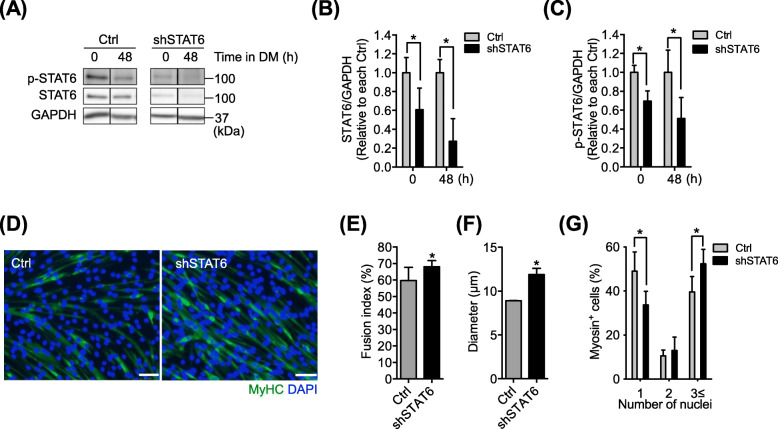
STAT6 knockdown improved myoblast fusion. Myoblasts were transfected with empty (Ctrl) or shSTAT6 vectors and grown in GM. After 48 h, the cells were harvested to ascertain p-STAT6, STAT6, and GAPDH levels via Western blotting. **a** representative images of Western blot bands. Relative protein expression levels of **b** STAT6 and **c** p-STAT6. *n* = 6 per group. *p < .05 vs. Ctrl. **d** representative immunostained myotubes positive for MyHC (green). Nuclei were stained with DAPI (blue). Scale bar = 50 μm. **e** Calculation of fusion index in the Ctrl and shSTAT6 treatment. *n* = 4. **f** Diameters of myotubes in the Ctrl and shSTAT6 treatments. **g** Percentages of myosin-positive cells. *n* = 4. *p < .05 vs. Ctrl. Data are presented as mean ± SD

Given that STAT6 deactivation is necessary during myotube formation, we hypothesized that the inhibition of STAT6 promotes myoblast fusion. To test this hypothesis, the myoblasts were transfected using a shSTAT6 vector to knockdown STAT6 and was maintained for 48 h in GM. Control myoblasts were transfected with an empty vector (i.e., Ctrl). After 48 h, the medium was switched to DM. Then incubation continued for 48 h. Based on Western blot (Fig. 3a), we confirmed that the expression of STAT6 (Fig. 3b) and of p-STAT6 (Fig. 3c) decreased significantly after shRNA transfection at 0 and 48 h of incubation in DM. We then conducted an immunohistochemical analysis to find the levels of myotube formation (Fig. 3d). The fusion index was modest but significantly higher in STAT6-knocked-down cells than in Ctrl cells (Fig. 3e). The diameter in STAT6-knocked-down cells was significantly higher than that in Ctrl cells (Fig. 3f). The percentage of myosin-positive cells possessing a single nucleus decreased, although the percentage of cells possessing three or more nuclei increased significantly in STAT6-knocked-down cells (Fig. 3g). Collectively, these results suggest an inhibitory role of STAT6 in myoblast fusion.

### STAT6 implicates gene expression related to myoblast fusion

**Fig. 4:**
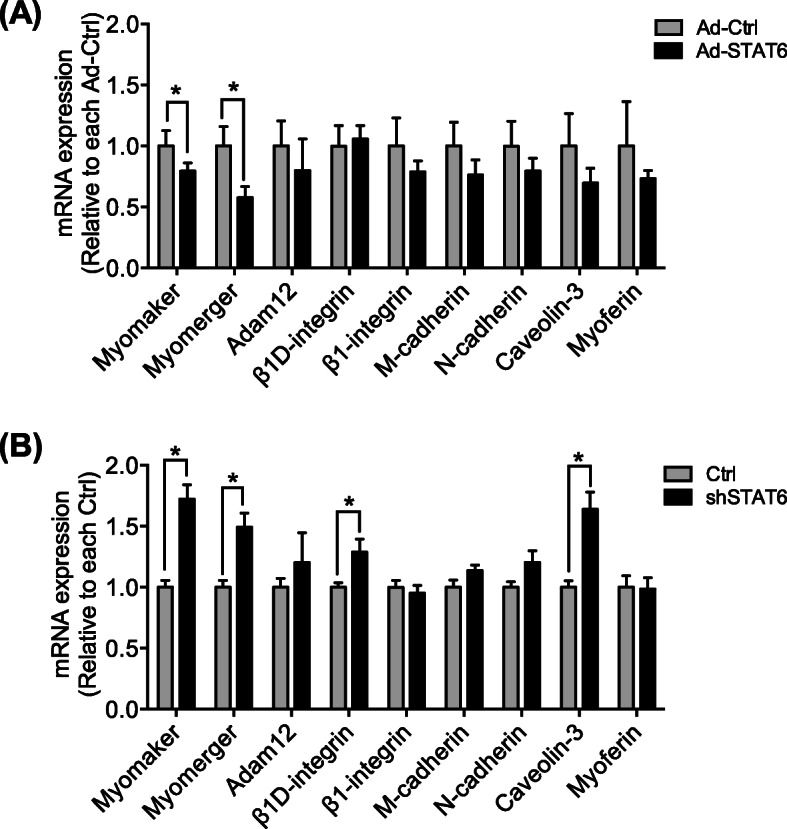
Expression of mRNA related to myoblast fusion in STAT6-overexpressed and STAT6-knockdown cells. **a** mRNA expression in the Ad-Ctrl and Ad-STAT6 treatments. *n* = 4 per group. *p < .05 vs. Ctrl. **b** mRNA expression in the Ctrl and shSTAT6 treatments. *n* = 6 per group. *p < .05 vs. Ctrl. Data are presented as mean ± SD

Several molecules are known to be associated with myoblast fusion. We next tested whether those fusion-related molecules might be affected by the experimental modulation of STAT6. The respective mRNA expressions of myomaker [28], myomerger [29], Adam12 [30], β1D-integrin [31], β1-integrin [32], M-cadherin [33], N-cadherin [34], caveolin-3 [35], and myoferin [36] were compared with myotubes differentiated for 48 h between Ad-Ctrl-infected and Ad-STAT6-infected cells. As portrayed in Fig. 4a, many molecules tended to be lower. Myomaker and myomerger were decreased significantly in Ad-STAT6 cells. By contrast, myomaker, myomerger, β1D-integrin, and caveolin-3 mRNAs were increased significantly in STAT6-knocked-down cells compared to those in Ctrl cells (Fig. 4b). These results were comparable to results of morphological analysis shown in Figs. 2 and 3.

### STAT6 affected the differentiation of myoblasts

**Fig. 5:**
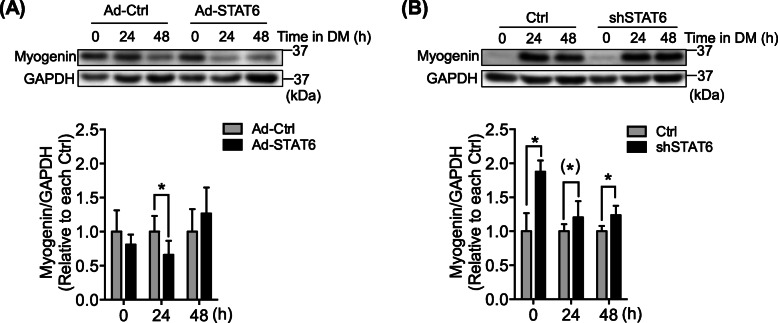
Intervention for STAT6 influences differentiation in culture. **a** Western blot for myogenin and GAPDH in the Ad-Ctrl and Ad-STAT6 treatments. *n* = 6. *p < .05 vs. Ad-Ctrl. **b** Western blot for myogenin and GAPDH in the Ctrl and shSTAT6 treatments. *n* = 6. *p < .05 vs. Ctrl. (*) *p* = .08 vs. Ctrl. Data are presented as mean ± SD

Because STAT6 manipulation was performed in myoblasts, we next investigated whether STAT6 is also involved in myoblast differentiation and proliferation as part of prefusion events. First, myogenin expression was analyzed during DM incubation for 0, 24, and 48 h in Ad-Ctrl and Ad-STAT6 infected cells. The adenoviral infection seemed to induce myogenin expression at 0 h, but the expression was found to have no significant difference between Ad-Ctrl and Ad-STAT6 (Fig. 5a). The myogenin expression was decreased significantly in Ad-STAT6 cells after 24 h of DM incubation (Fig. 5a). No difference was found at 48 h (Fig. 5a). On the other hand, at 0 and after 48 h of DM incubation, myogenin expression was increased significantly in STAT6-knocked-down cells compared to in Ctrl cells (Fig. 5b). A similar result was obtained at 24 h (*p* = .08). Although the time course change varied between gain-of-function and loss-of-function conditions, these results suggest that STAT6 was implicated in myogenin expression as an inhibitory factor during DM incubation. We next analyzed the differentiation index in an identical sample used for fusion index analysis in Figs. 2 and 3. Results show that the differentiation index was decreased significantly in Ad-STAT6 myotubes (Fig. S1A) but that it was increased significantly in STAT6-knocked-down myotubes (Fig. S1B). Considered collectively, these results suggest that STAT6 plays inhibitory roles in the differentiation program.

We also tested the effects of overexpression of STAT6 on myoblast proliferation. Results show no difference in the indirect cell number or proliferation rate between Ad-Ctrl and Ad-STAT6 myoblasts (Fig. S2A–S2C). Therefore, STAT6 was not suggestive of implicating myoblast proliferation.

### STAT6-KO mice exhibit improved regeneration after injury

**Fig. 6:**
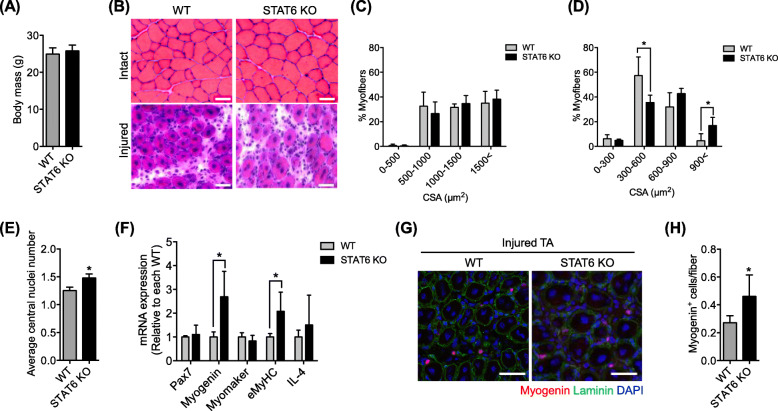
STAT6 knockout (KO) in mice improved muscle regeneration after injury. **a** Body mass in WT and STAT6-KO mice. **b** Representative image of hematoxylin and eosin-stained intact TA muscle and regenerating TA muscle sampled at 5 days after injury from WT and STAT6-KO mice. Scale bar = 50 μm. Size distribution of myofiber cross-sectional area (CSA) in intact TA muscle (**c**) and injured TA muscle (**d**). **e** Average central nuclei number in myofiber of regenerating TA muscle. *n* = 5. *p < .05 vs. WT. **f** mRNA expression in injured TA muscle. *n* = 5. *p < .05 vs. WT. **g** Representative image of myogenin staining in injured TA. **h** Quantification of myogenin^+^ cells per myofiber in injured TA. *n* = 5. *p < .05 vs. WT. Data are presented as mean ± SD

We examined whether the absence of STAT6 affects adult regenerative myogenesis in vivo, or not*.* No difference was found in the body mass of WT or STAT6-KO mice studied (Fig. 6a). Cardiotoxin in saline was injected into the left TA muscles of WT and STAT6-KO mice to induce muscle injury, followed by regeneration. At 5 days post-injection, no difference was found in fiber CSA distribution in the right intact TA (Fig. 6b and c). However, in the regenerated TA, the percentage of myofibers between 300 and 600 μm was significantly lower, whereas the percentage of myofibers of more than 900 μm was significantly higher in STAT6-KO than in WT mice (Fig. 6b and d). The average number of central nuclei in regenerating myofibers of the TA muscle was found to be significantly higher in STAT6-KO than in WT mice (Fig. 6e). Next, mRNA expression of pax7, myogenin, myomaker, embryonic MyHC (eMyHC) , and IL-4 was examined in the regenerated TA muscle. No change in pax7 or myomaker mRNA, myogenin or eMyHC mRNA was significantly higher in STAT6-KO than in WT mice (Fig. 6f). No difference was found in IL-4 mRNA between WT and STAT6-KO mice (Fig. 6f). We also made a cryosection of TA to examine myogenin^+^ cell numbers using immunohistochemistry. Myogenin^+^ cell numbers were significantly higher in STAT6-KO mice than in WT mice (Fig. 6g and h). Altogether, these results indicate that muscle regeneration after injury was facilitated in STAT6-KO mice by enhancing myogenic differentiation and fusion. The comparable IL-4 mRNA levels imply that the effects of IL-4 were comparable between WT and STAT6-KO mice irrespective of their regeneration discrepancy.

In a separate analysis, we examined pax7^+^ satellite cell numbers immunohistochemically (Fig. S3A). We found no difference between the numbers obtained for WT and STAT6-KO mice (Fig. S3B). In light of the results of cell number analysis shown in Fig. S2, we infer that STAT6 does not influence myogenic cell proliferation.

### Inhibitory action of STAT6 is independent of IL-4 signaling

**Fig. 7:**
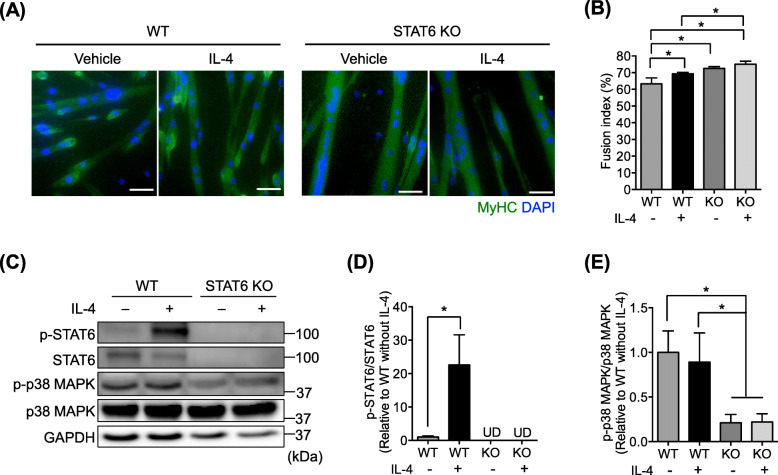
IL-4 and STAT6 independently regulated myogenesis. Myoblasts were isolated from WT and STAT6-KO mice and maintained in DM with or without IL-4 treatment. **a** Representative immunostained myotubes positive for MyHC (green). Nuclei were stained with DAPI (blue). Scale bar = 50 μm. **b** Quantification of fusion index. *n* = 5. *p < .05. **c** Representative protein bands for p-STAT6, STAT6, p-p38 MAPK, p38 MAPK, and GAPDH. **d** p-STAT6 protein expression. *n* = 6. *p < .05 by unpaired *t* test. UD denotes undetected. **e** p-p38 MAPK protein expression. *p < .05. Data are presented as mean ± SD

Using the STAT6-KO mice model, we then sought to elucidate whether STAT6 can mediate IL-4-induced stimulation of myotube formation. The myoblasts isolated from WT and STAT6-KO mice were differentiated by DM incubation for 48 h with or without IL-4. Then, the myotubes were fixed and stained with MyHC and DAPI to examine the fusion index (Fig. 7a). Results indicate that IL-4 significantly increased the fusion index in WT cells (Fig. 7b). The fusion index in STAT6-KO cells was significantly higher than that in WT cells irrespective of IL-4 treatment (Fig. 7b). It is particularly interesting that the fusion index in STAT6-KO cells with IL-4 treatment was significantly higher than that in WT cells with IL-4 treatment. These results suggest that IL-4 and STAT6 independently implicate myotube formation. This finding implies that STAT6 did not mediate a positive role of IL-4 in myotube formation.

To ascertain the reasons for the separate influence of IL-4 and STAT6, we sought candidate molecules for regulating myogenesis via STAT6. During this process, we found a discrepancy in p38 MAPK, which is an essential kinase for myogenesis [37], in those cells by Western blot (Fig. 7c). As expected, IL-4 increased p-STAT6 in WT cells significantly (Fig. 7d). Also, STAT6 was not detected in STAT6-KO cells (Fig. 7d). In this situation, p-p38MAPK in STAT6-KO cells was significantly decreased compared to that in WT cells, irrespective of IL-4 treatment (Fig. 7e). These results implicate p38 MAPK in STAT6 during myotube formation. Considering the results obtained for the fusion index (Fig. 7b), that the repressive role of STAT6 in myotube formation is mediated by p38 MAPK activity, independent of IL-4 action.

### Myoblast–myotube fusion is attenuated in STAT6-overexpressed myoblasts

**Fig. 8:**
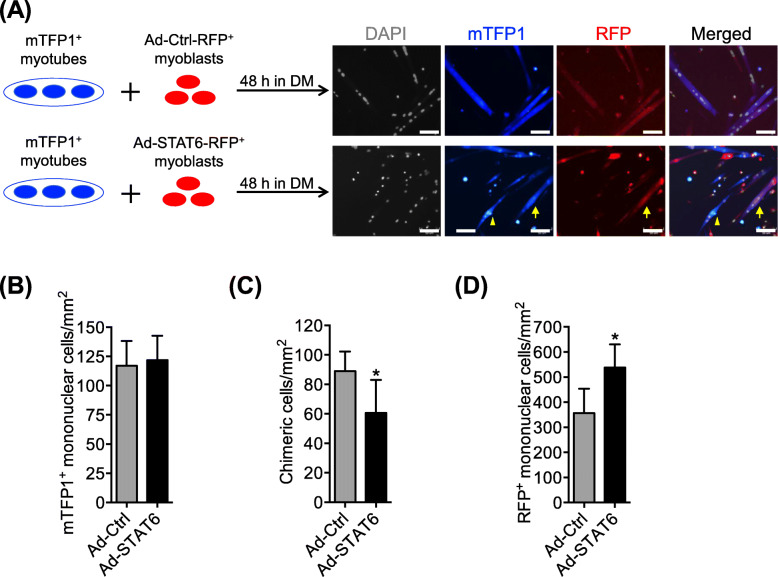
STAT6 overexpression in myoblasts impairs its fusion with myotubes. **a** Representation of cell mixing assay performed to examine fusion between myoblast and myotubes. Representative images show myotube formation in individual and mixed cultures. Arrows indicate myotubes fused with RFP^+^ myoblasts. Arrowheads indicate myotubes that are not fused with RFP^+^ myoblasts. Scale bar = 50 μm. Numbers of **b** mTFP1^+^ and **c** chimeric cells and **d** adenovirus-infected RFP^+^ cells per field in mixed cultures. *n* = 6 for each treatment. *p < .05 vs. Ad-Ctrl treatment. Data are presented as mean ± SD

We further examined the possibility of an unlikely mediation of STAT6 in IL-4-induced myotube formation. It has been established that IL-4 signaling is important for myoblasts to fuse with myotubes [21]. Therefore, it would be expected that myoblast–myotube fusion can be enhanced by STAT6 activation if the IL-4-STAT6 axis served as a stimulatory pathway for it. To this end, we followed an earlier reported protocol but used STAT6 overexpression instead of IL-4Rα KO in myoblasts [21]. Isolated mTFP1^+^ myoblasts expressing STAT6 normally were pre-incubated in DM for 48 h to form mTFP1^+^ myotubes. Concomitantly, the WT myoblasts were infected with either Ad-STAT6 or Ad-Ctrl expressing RFP. These RFP^+^ myoblasts were then added to mTFP1^+^ myotubes and were maintained for the next 48 h to induce fusion of RFP^+^ myoblasts to mTFP1^+^ myotubes (Fig. 8a). The numbers of mTFP1^+^ mononuclear (i.e., unfused) cells were comparable between Ad-STAT6 and Ad-Ctrl cultures (Fig. 8b), suggesting that their myotube conditions were comparable. The chimeric myotubes, expected mostly to signify the fusion of adenovirus-infected myoblasts with myotubes, were significantly fewer in the Ad-STAT6 treatment (Fig. 8c). The number of unfused RFP^+^ mononuclear cells was significantly higher in the Ad-STAT6 than in the Ad-Ctrl culture (Fig. 8d). Therefore, although this experimental procedure can not entirely eliminate the possibility of myoblast–myoblast fusion, results seem to support the notion that STAT6 does not mediate IL-4-linked promotion of myoblast fusion.

## Discussion

**Fig. 9:**
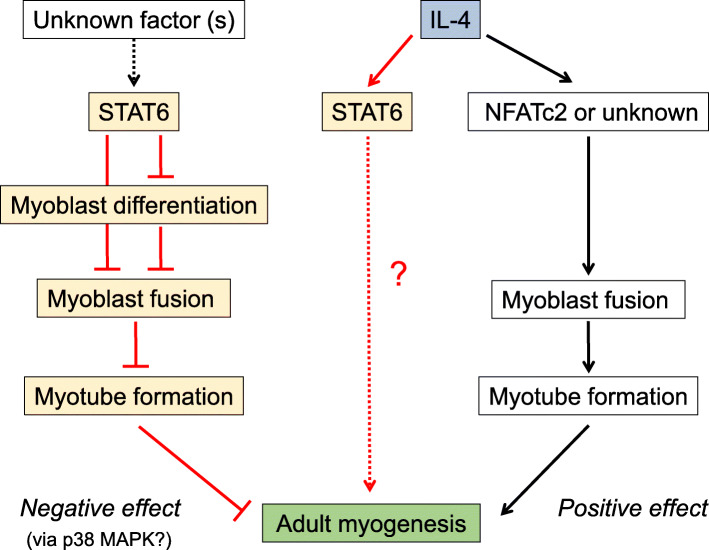
Putative roles of STAT6 in adult myogenesis. IL-4 can activate STAT6 in myotubes. However, STAT6 inhibits myotube formation. IL-4 improves myoblast fusion independently of STAT6 activity. The IL-4-STAT6 signaling axis therefore does not account for IL-4 related myotube formation processes. The red line is based on results from this study. The black line shows data referred from earlier studies. The dashed line represents unvalidated data

Mechanisms underlying myoblast differentiation and fusion remain unclear. Our first-time gain-of-function and loss-of-function experiments demonstrated that overexpression of STAT6 inhibits, whereas excessive inhibition of STAT6 improves, myoblast differentiation and fusion in vitro. In vivo experiments using STAT6-KO mice demonstrated that the muscle regeneration process was improved in the absence of STAT6 expression. Results also demonstrate that inhibition of myogenesis by STAT6 might not be associated with IL-4. These results suggest that STAT6 is a protein that negatively regulates adult myogenesis. Figure 9 depicts a putative scheme of these study results.

Results of earlier studies have shown that IL-4 facilitates fusion in cultured myocytes [19, 21] or that IL-4 promotes myogenic differentiation in colon carcinoma-bearing mice [38]. Earlier studies in nonmuscle cell types have demonstrated that STAT6 is activated primarily by IL-4 [12, 13]. Therefore, we speculated that STAT6 is involved in the muscle regeneration process as a positive regulator. However, although we found that IL-4 can activate STAT6 during myogenesis, results showed that STAT6 had an inhibitory rather than a stimulatory effect on muscle formation. Indeed, STAT6 activity was decreased during DM incubation. Results suggest that the deletion of STAT6 and IL-4 treatment independently improved the fusion index in culture. Moreover, STAT6-overexpressed myoblasts showed a lower capacity to fuse with myotube under culture conditions. In addition, whereas IL-4 KO and IL-4Rα KO mice did not exhibit improved CSA at 8 days of post injury [21], we observed a greater CSA at 5 days post injury in STAT6-KO mice than in WT mice. Altogether, these findings suggest that the IL-4-STAT6 signaling axis is not responsible for myotube formation. Other mechanisms are expected to control STAT6 activity to attenuate myogenesis. Based on results of earlier studies, multiple molecules are potentially involved in myogenesis, which can regulate STAT6 in cellular events. For instance, the mammalian target of rapamycin (mTOR) inhibits STAT6 activity in T cells [39]. Also, mTOR-signaling activation is important for myogenesis [40, 41]. Consequently, mTOR activation might inhibit STAT6 signaling during myotube formation. Alternatively, interferon-β can activate STAT6 in hepatoma cells [42]. Because interferon-β impairs myotube formation [43], interferon-β signaling might negatively regulate myogenesis via STAT6. Some molecules such as interferon-α [42, 44], IL-13 [45], and leptin [46] interact with STAT6 in nonmuscle cell types. Eventually, further study will be necessary to identify molecules that control STAT6 activity in myogenesis as an inhibitory factor.

We observed an IL-4-independent decrease in p38 MAPK activity in fusion-facilitated STAT6-KO myotubes. This decrease suggests a possible relation between STAT6 and p38 MAPK during myogenesis. Actually, the role of p38 MAPK in myogenesis is complicated: p38 MAPK is necessary to execute timely myogenic differentiation by activating myocyte enhancer factor 2 (MEF2) [47] or MyoD and its co-molecule E47 [37]. Also, p38 MAPK mediates inhibition of myogenic cell cycling as a molecular switch to regulate myogenic commitment [48]. These results suggest that p38 MAPK contributes to advancement of myotube formation. By contrast, an inhibitory role of p38 MAPK in myogenesis has also been suggested. Suelves et al. has demonstrated that inhibition of p38 MAPK activity increased desmin and α-actin expression in C2C12 myoblast differentiated for 5 days [49]. Contrary to an earlier report by Llouis et al. [37], results of at least one study show that p38 MAPK might mediate the inhibition of E47 activity by mitogen and extracellular kinase kinase 1 during differentiation in C2C12 cells [50]. Moreover, Weston et al. has shown that p38 MAPK inhibition activated myogenin promoter and increased myogenin and MEF2C gene expression in C2C12 cells [51]. They also demonstrated that p38 MAPK inhibition promoted myogenesis in the distal limb or proximal mesenchyme myoblasts [51]. Accordingly, findings obtained from the current study agree with those of the earlier study [52] because STAT6 inhibition or deletion stimulated differentiation and fusion with attenuated p38 MAPK activity. A specific link between STAT6 and p38 MAPK during myogenesis must be clarified.

Although our STAT6-KO animals exhibited interesting results, attention must be devoted to their interpretation. First, our mice lacked STAT6 globally and congenitally. Therefore, we cannot rule out the possibility of physiological compensation for the loss of STAT6. Moreover, because STAT6 is necessary for immune cell regulation [12, 13], some alteration in the systemic inflammatory situation can be expected. However, results from acute shRNA-based STAT6 knockdown in isolated and STAT6-KO myoblasts experiments partially counter those concerns. Second, no significant difference was found in cross-sectional area between WT and STAT6-KO intact muscles, implying no developmental problem in the mice. Considering that STAT6 influences myogenic differentiation, it is expected that developmental defects would occur in STAT6-KO mice because considerable myogenesis occurs in the embryo. Although we currently have no explanation related to this point, it is noteworthy that molecular machinery might be largely shared but not be completely identical between de novo developmental and adult myogenesis [53–55].

The physiological significance of STAT6 inhibitory function during myogenesis remains unknown. One supposition is that STAT6 adjusts the proper timing of myoblasts for myogenic commitment. Myogenesis is tightly regulated by the sequential activation or deactivation of signaling cascades [5, 52]. The dysregulation of the cascades impairs myofiber formation. Our results demonstrate that STAT6 activity was evident for predifferentiation. Then, activity levels decreased along with the induction of differentiation. We observed that STAT6 can affect myogenin expression. Consequently, STAT6 might prevent the progression of myoblast differentiation and fusion until the myoblasts enter a fusion-competent state. At this time, fusion is allowed to proceed by decreasing STAT6 activity. Considering this inference as true, our findings related to myogenesis promotion by STAT6 inhibition imply a harmful influence of intact myofiber formation under practical situations. Consequently, the influence of STAT6 inhibition over the entire adult myogenesis period, during which a mature myofiber is established, must be elucidated.

## Conclusion

Results indicate that STAT6 plays an inhibitory role in myoblast differentiation and fusion in adults. Moreover, the results suggest that the facilitation of myotube formation by IL-4 is independent of STAT6. Consequently, STAT6 might be a clinical target to achieve efficient muscle formation.

## Supplementary Information

**Additional file 1: Figure S1**. Differentiation index in STAT6-overexpressed and STAT6-inhibited cells. (A) Differentiation index in the Ad-Ctrl and Ad-STAT6 treatments. *n* = 5. *p < .05 vs. Ad-Ctrl. Images in Fig. 2 were used for analysis. (B) Differentiation index in the Ctrl and shSTAT6 treatments. *n* = 5. *p < .05 vs. Ctrl. Images in Fig. 3 were used for analysis. Data are presented as mean ± SD. **Figure S2**. Proliferation of STAT6-overexpressed myoblasts. (A) Representative bright-field images of myoblasts in Ad-Ctrl and Ad-STAT6 cells. Scale bar = 50 μm. (B) Absorbance at 450 nm in Ad-Ctrl and Ad-STAT6 cells using a CCK cell counting kit. (C) Proliferation rate in Ad-Ctrl and Ad-STAT6 cells. *n* = 6. Data are presented as mean ± SD. **Figure S3**. Pax7-positive cells in regenerating TA muscle of WT and STAT6-KO mice. (A) Representative images in CTX-injured TA muscle. Scale bar = 50 μm. (B) Quantification of pax7^+^ cells per myofiber. *n* = 5. Data are presented as mean ± SD. **Supplementary Table S1**. Primer sequences for QRT-PCR.

## Data Availability

All data generated and analyzed during the study are available from the corresponding author upon reasonable request.

## Abbreviations

ANOVA: Analysis of variance
DAPI: 4′, 6-Diamidino-2-phenylinodole
DM: Differentiation medium
ECL: Enhanced chemiluminescence
GAPDH: Glyceraldehyde-3-phosphate dehydrogenase
GM: Growth medium
IL: Interleukin
MAPK: Mitogen-activated protein kinase
KO: Knockout
mTFP1: Monomeric teal fluorescent protein 1
PVDF: Poly-vinylidene di-fluoride
RFP: Red fluorescent protein
SDS-PAGE: Sodium dodecyl sulfate – polyacrylamide gel electrophoresis
shRNA: Short hairpin RNA
siRNA: Small interfering RNA
STAT: Signal transducer and activator of transcription
