# Supplementary material for: Transcription factor signal transducer and activator of transcription 6 (STAT6) is an inhibitory factor for adult myogenesis
Source: Skelet Muscle. 2021 May 29;11:14. doi: 10.1186/s13395-021-00271-8 (PMC8164270; doi:10.1186/s13395-021-00271-8)
Supplement: Supplementary file 1 — Additional file 1: Figure S1. Differentiation index in STAT6-overexpressed and STAT6-inhibited cells. (A) Differentiation index in the Ad-Ctrl and Ad-STAT6 treatments. n = 5. *p < .05 vs. Ad-Ctrl. Images in Fig. 2 were used for analysis. (B) Differentiation index in the Ctrl and shSTAT6 treatments. n = 5. *p < .05 vs. Ctrl. Images in Fig. 3 were used for analysis. Data are presented as mean ± SD. Figure S2. Proliferation of STAT6-overexpressed myoblasts. (A) Representative bright-field images of myoblasts in Ad-Ctrl and Ad-STAT6 cells. Scale bar = 50 μm. (B) Absorbance at 450 nm in Ad-Ctrl and Ad-STAT6 cells using a CCK cell counting kit. (C) Proliferation rate in Ad-Ctrl and Ad-STAT6 cells. n = 6. Data are presented as mean ± SD. Figure S3. Pax7-positive cells in regenerating TA muscle of WT and STAT6-KO mice. (A) Representative images in CTX-injured TA muscle. Scale bar = 50 μm. (B) Quantification of pax7+ cells per myofiber. n = 5. Data are presented as mean ± SD. Supplementary Table S1. Primer sequences for QRT-PCR. [file 13395_2021_271_MOESM1_ESM.pptx]

## Slide 1
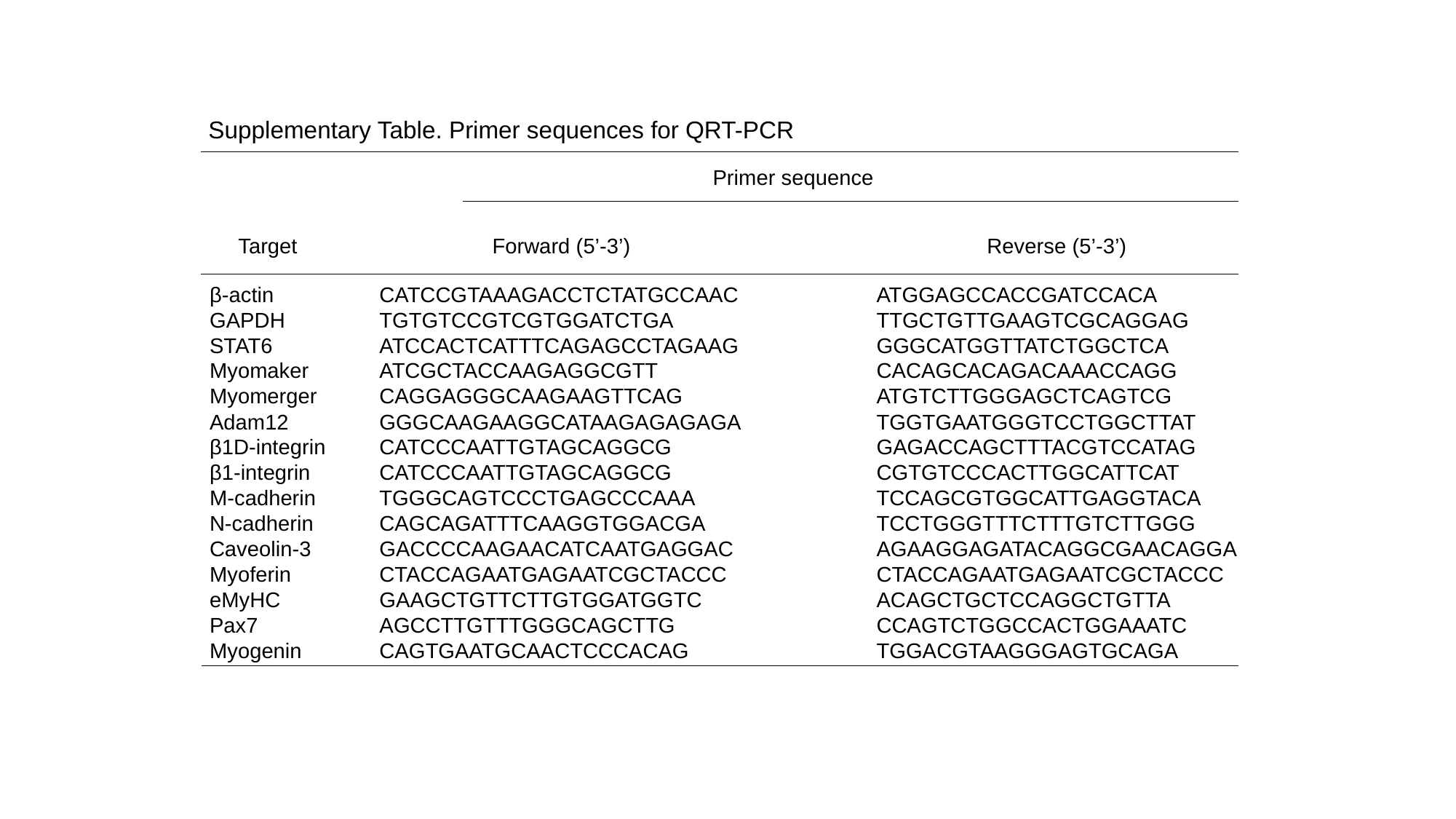

Supplementary Table. Primer sequences for QRT-PCR
Primer sequence
Target
Forward (5’-3’)
Reverse (5’-3’)
β-actin
GAPDH
STAT6
Myomaker
Myomerger
Adam12
β1D-integrin
β1-integrin
M-cadherin
N-cadherin
Caveolin-3
Myoferin
eMyHC
Pax7
Myogenin
CATCCGTAAAGACCTCTATGCCAAC
TGTGTCCGTCGTGGATCTGA
ATCCACTCATTTCAGAGCCTAGAAG
ATCGCTACCAAGAGGCGTT
CAGGAGGGCAAGAAGTTCAG
GGGCAAGAAGGCATAAGAGAGAGA
CATCCCAATTGTAGCAGGCG
CATCCCAATTGTAGCAGGCG
TGGGCAGTCCCTGAGCCCAAA
CAGCAGATTTCAAGGTGGACGA
GACCCCAAGAACATCAATGAGGAC
CTACCAGAATGAGAATCGCTACCC
GAAGCTGTTCTTGTGGATGGTC
AGCCTTGTTTGGGCAGCTTG
CAGTGAATGCAACTCCCACAG
ATGGAGCCACCGATCCACA
TTGCTGTTGAAGTCGCAGGAG
GGGCATGGTTATCTGGCTCA
CACAGCACAGACAAACCAGG
ATGTCTTGGGAGCTCAGTCG
TGGTGAATGGGTCCTGGCTTAT
GAGACCAGCTTTACGTCCATAG
CGTGTCCCACTTGGCATTCAT
TCCAGCGTGGCATTGAGGTACA
TCCTGGGTTTCTTTGTCTTGGG
AGAAGGAGATACAGGCGAACAGGA
CTACCAGAATGAGAATCGCTACCC
ACAGCTGCTCCAGGCTGTTA
CCAGTCTGGCCACTGGAAATC
TGGACGTAAGGGAGTGCAGA

## Slide 2
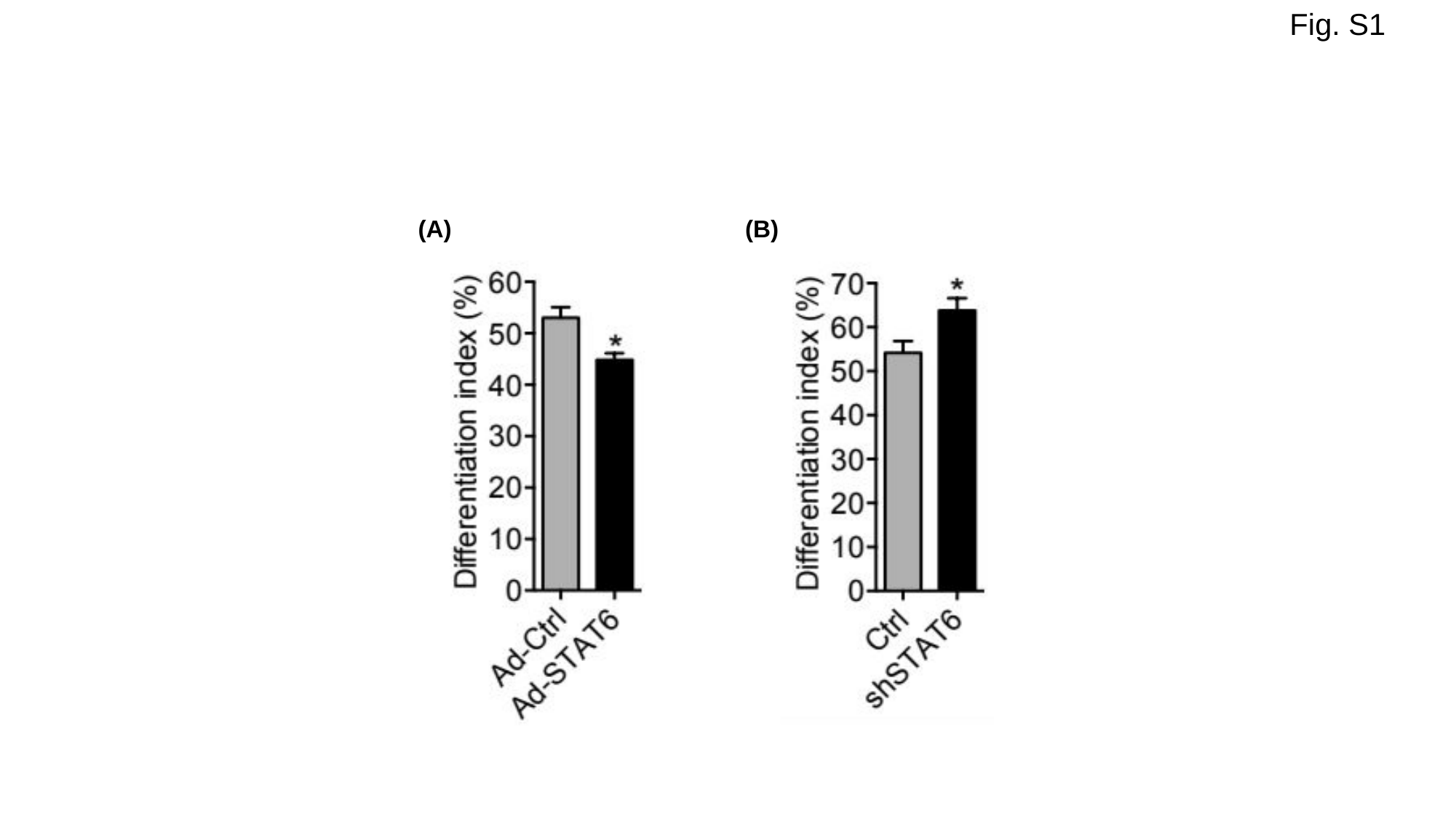

Fig. S1
(A)
(B)

## Slide 3
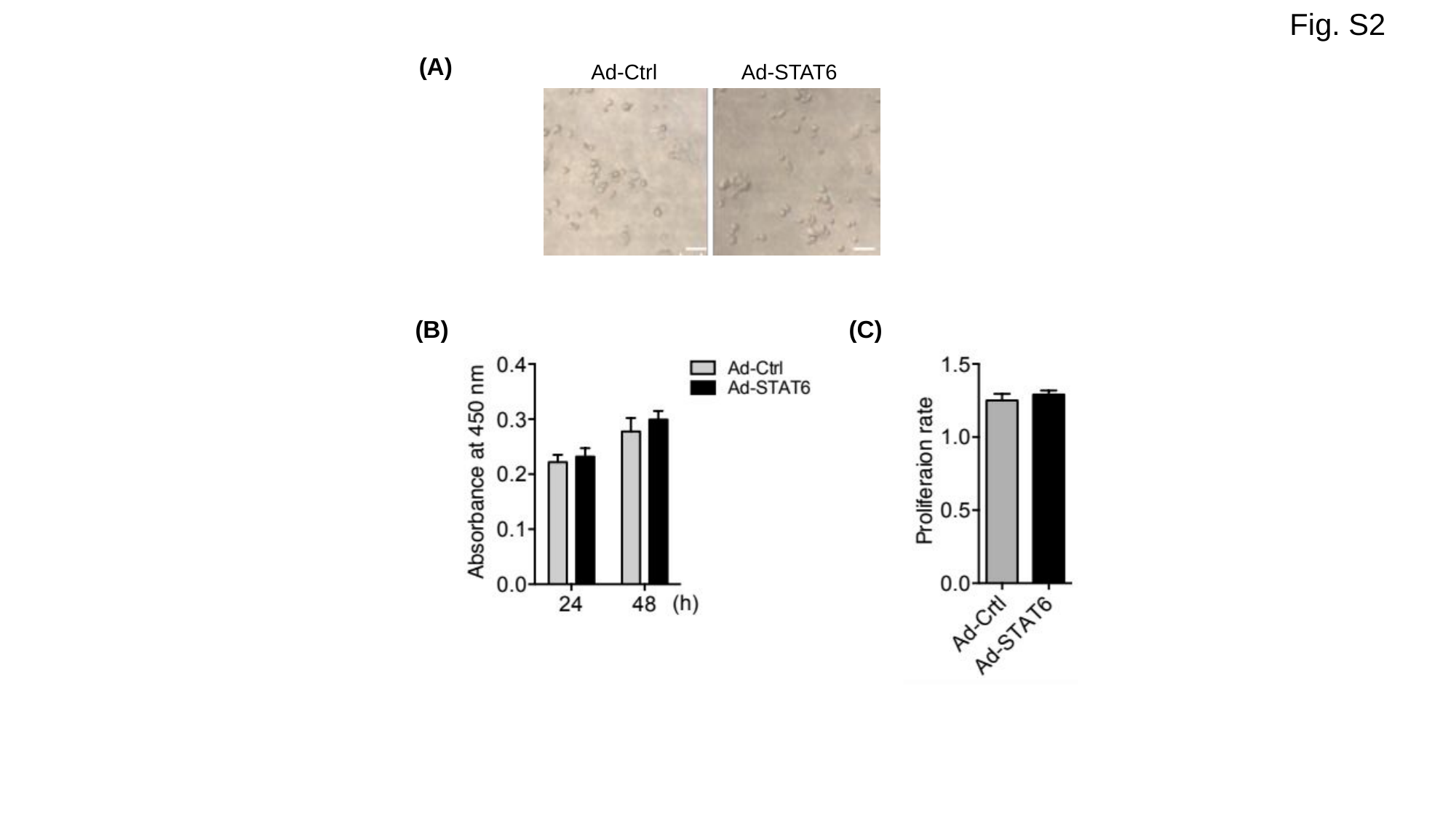

Fig. S2
(A)
Ad-Ctrl
Ad-STAT6
(C)
(B)

## Slide 4
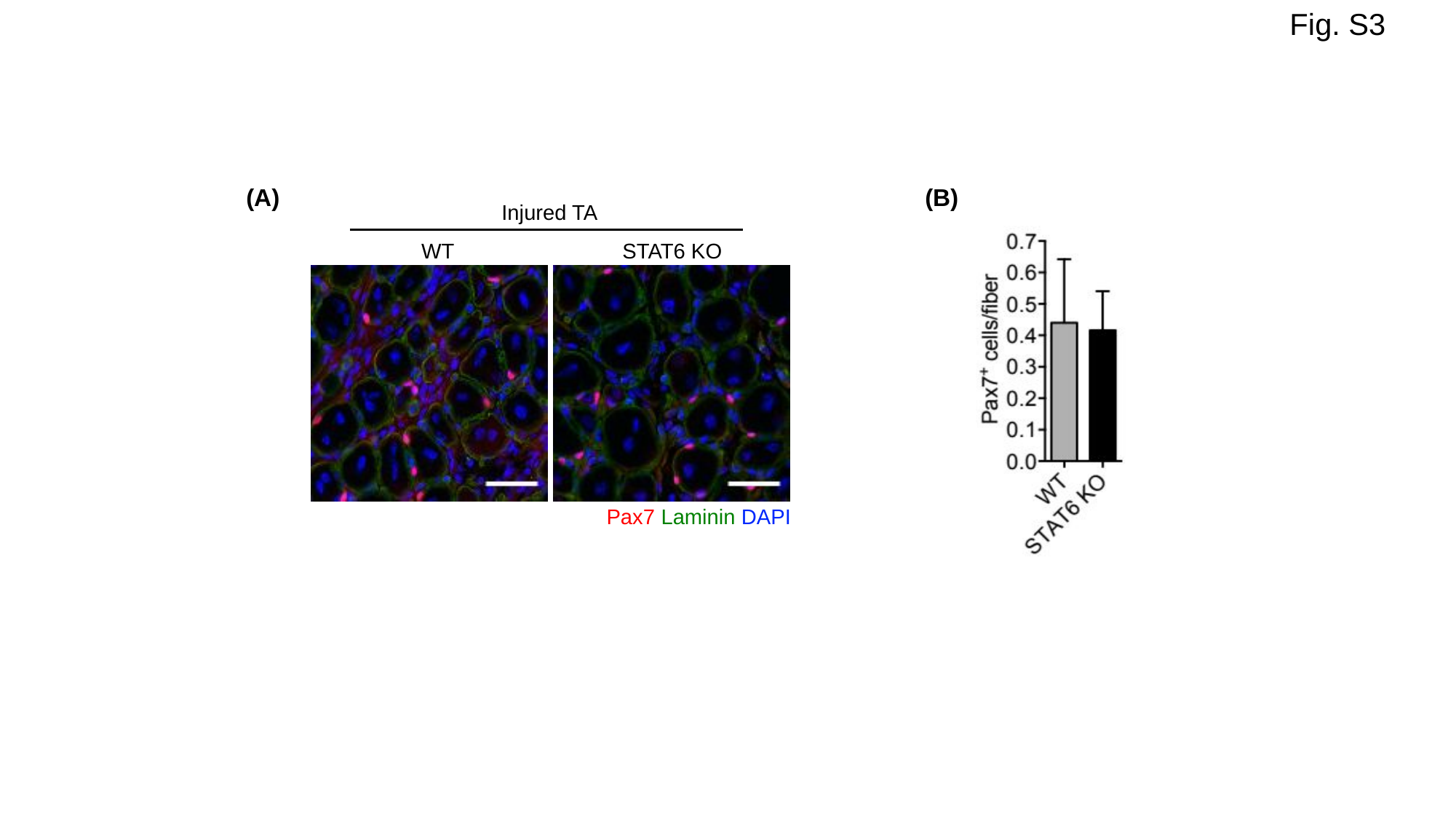

Fig. S3
(A)
(B)
Injured TA
WT
STAT6 KO
Pax7 Laminin DAPI
